# Stereotactic body radiotherapy improves the survival of patients with oligometastatic non‐small cell lung cancer

**DOI:** 10.1002/cam4.2366

**Published:** 2019-06-27

**Authors:** Wen Ouyang, Jing Yu, Shuake Nuerjiang, Zhijun Li, Dajiang Wang, Xiaoyong Wang, Junhong Zhang, Conghua Xie

**Affiliations:** ^1^ Department of Radiation and Medical Oncology Zhongnan Hospital of Wuhan University Wuhan China; ^2^ Hubei Key Laboratory of Tumor Biological Behaviors Zhongnan Hospital of Wuhan University Wuhan China; ^3^ Hubei Clinical Cancer Study Center Zhongnan Hospital of Wuhan University Wuhan China

**Keywords:** non‐small cell lung cancer, oligometastases, stereotactic body radiotherapy

## Abstract

**Purpose:**

The aim of the study was to evaluate the efficacy and safety of stereotactic body radiotherapy (SBRT) for pulmonary lesions in oligometastatic non‐small cell lung cancer (NSCLC) patients, to explore prognostic factors of progression‐free survival (PFS) and overall survival (OS), to validate improved survival contributed by SBRT in oligometastatic NSCLC patients.

**Patients and methods:**

A total of 71 oligometastatic NSCLC patients with 86 pulmonary lesions treated with SBRT in our institute between 2012 and 2018 were included. Local control (LC), progression‐free survival (PFS), and overall survival (OS) were calculated using Kaplan‐Meier method. Prognostic factors of PFS and OS were analyzed using univariate and multivariate Cox analyses. Subgroup analyses were performed to investigate the impact of SBRT on PFS and OS during first line systemic treatment.

**Results:**

After a median follow‐up of 17.6 months, 2‐year LC and OS rates were 82.6% and 55.3%, respectively. No grade 4 or more toxicities were observed. Multivariate analysis showed systemic treatment regimen before SBRT was an independent prognostic factor of PFS, but not for OS. Among this cohort, patients receiving first line target therapy could show a better PFS and OS than those undergoing first line chemotherapy (target therapy vs chemotherapy, PFS, 26.4 m vs 6.9 m; OS, 34.8 m vs 15.5 m).

**Conclusions:**

SBRT for pulmonary lesions was a feasible and tolerable option for oligometastatic NSCLC patients. Delivery of SBRT for pulmonary lesions improved outcomes of oligometastatic NSCLC patients. Finally, SBRT combined with first line target therapy might have optimal outcomes.

## 1 INTRODUCTION

The standard therapy for patients with metastatic non‐small cell lung cancer (NSCLC) was systemic treatment with palliative intent, and the role of local treatment remains controversial.^1^ Oligometastases, which was established by Hellman and Weichselbaum in 1995, is an intermediate state between locoregional tumor spread and disseminated metastases.^2^ Oligometastases defined as limited metastases (≤5) reflects a moderate risk of distant metastasis, which could benefit from local therapy.^3^, ^4^, ^5^, ^6^ Indeed, retrospective studies of pulmonary and hepatic metastasectomy from different primary tumors showed a 5‐year survival of 20%‐47%.^7^, ^8^, ^9^, ^10^, ^11^, ^12^

Stereotactic body radiation therapy (SBRT) is a highly conformal and hypofractionated radiotherapy. Because peripheral lung tissue obeys the parallel architecture model of radiobiology, high‐dose radiation can be focally administered without excessive risk of radiation‐induced pneumonitis (RP), provided sufficient normal lung can be spared. Historically, SBRT is a non‐surgical alternative treatment for elderly patients with early‐stage NSCLC who are inoperable due to comorbidities or insufficient pulmonary function.^13^, ^14^ Several prospective phase II trials of SBRT showed excellent outcomes for I stage NSCLC with 3‐year overall survival (OS) and local control (LC) rates of 56%‐60% and 85%‐98%, respectively.^15^, ^16^, ^17^ Based on experiences in primary early‐stage NSCLC, SBRT has also been introduced in the treatment of pulmonary oligometastases from various primary tumors. However, current scientific data on the outcomes of SBRT for pulmonary oligometastases are characterized by small patient cohorts and heterogeneous populations. Up to now, rare studies report the efficacy and safety of SBRT treating for pulmonary lesions of oligometastatic NSCLC patients.

Retrospective analyses of failure patterns after first line systemic therapy for metastatic NSCLC show that most of the progression events occur only at sites of known disease at baseline, rather than at new sites.^18^ It is believed that local treatment for those metastases may reduce the burden of tumor or remove dominant disease sites that may seed other sites in the future. Consequently, local treatment of metastatic disease following systematic treatment contributes to an improved progression‐free survival (PFS).^3^, ^4^, ^5^, ^6^ However, information on the oligometastatic NSCLC cases in which SBRT for pulmonary lesions may improve the survival remains insufficient and the selection of appropriate cases for SBRT is of particular concern to oncologists.

Therefore, we reviewed the data of 71 oligometastatic NSCLC patients treated with SBRT in our institution, to evaluate the outcomes, and to explore the potential prognostic factors.

## 2 PATIENTS AND METHODS

### 2.1 Patients

Between January 2012 and June 2018, 71 oligometastatic NSCLC patients with a total of 86 pulmonary lesions, were treated with hypofrationated SBRT at the Department of Radiation and Medical Oncology of Zhongnan hospital of Wuhan University, and were reviewed on the basis of electronic medical records. This retrospective study was approved by the Ethics Committee of Zhongnan hospital of Wuhan University. The ethics committee approved oral informed consent, as the data were reviewed and analyzed anonymously. Informed consent was obtained orally from the included patients by telephone. Included patients were those who met the following criteria: (a) A performance status (PS) of 0‐2; (b) Patients with one to two pulmonary lesions, which were diagnosed by biopsy or clinical on the basis of CT ± FDG‐PET imaging; (c) Patients with total metastases limited to five lesions (termed curative); (d) After system therapy, the target lesions were stable; (e) Patients with disease control after initial systemic therapy; (f) Prior systemic therapy continued until the time of progression; (g) Follow‐up of more than 3 months. The clinical characteristics of the included patients are shown in Table 1.

**Table 1:** Clinical characteristics

Characteristic	No.	%
No. of patients	71	
No. of lesions	86	
ECOG score		
0‐1	65	91.5
2	6	8.5
Age, y	71	
≤70	49	69
>70	22	31
Median(Range)	67(33‐85)	
Gender	71	
Male	19	26.9
Female	52	73.1
Histology	71	
Squamous carcinoma	15	21.1
Adenocarcinoma	44	62
Others	5	7
Unkown	7	9.9
BMI	59	
Mean(95%CI)	22.65 (19.4‐25.9)	
Smoking	71	
Yes	29	40.8
No	42	59.2
CEA (ng/mL)	68	
Median(Range)	4.89 (1‐632)	
Number of metastases (all sites)	71	
1	24	33.8
2	25	35.2
3	21	29.2
4	1	1.4
Systemic treatment before SBRT	71	
None	19	21.1
EGFR‐TKI	37	52.1
Chemotherapy	15	21.1
Time interval between systemic treatment initiation and SBRT	71	
Median(Range,months)	6.2 (0‐57.4)	
Prior systemic therapy lines for metastatic disease	71	
No treatment before SBRT	22	31
First line treatment before SBRT	40	56.3
Second line treatment before SBRT	7	9.9
Third line treatment before SBRT	2	2.8

### 2.2 Treatment

All patients had raised both upper arms and were immobilized in the supine position. Limitation of respiratory motion was achieved by using abdominal compression, and computed tomography (CT) scans were obtained using a CT simulator (Siemens, German) with 3 mm slice thickness to recognize tumor localization and for dose calculation. The gross target volume (GTV) was contoured on lung windows and included only solid mass without ground‐glass changes. A planning target volume (PTV) was generated by expanding 5 mm radial and 10 mm craniocaudal margin of GTV. If 4DCT system was used, an internal target volume (ITV) was generated to encompass the internal motion of GTV in all respiratory phases. For these patients, the PTV was generated by expanding 5 mm around the ITV according to RTOG 0813. All dosimetry was performed with the goal of covering at least 95% of the PTV with the 100% isodose line and ensuring that all hot spots were within the GTV. Dose constraints for normal organs were taken from published trial RTOG 0618.

All treatments were calculated using a superposition/convolution algorithm on the CMS XiO (Varian, USA) treatment planning system (TPS) (before December 2015) and Tomotherapy (HT) TPS (Accuray, CA) (since December 2015), respectively. IMRT plans were universally multiple noncoplanar. HT plans were delivered using helical arcs. Daily image guidance, using either orthogonal x‐rays or on‐board CT imaging to relocate the target lesion before treatment delivery. The median dose was 50Gy in 5 fractions. The median biologically effective dose (BED), assuming an α/β ratio of 10 Gy (BED10), was 100Gy. The characteristics of lesions and treatments are summarized in Table 2.

**Table 2:** SBRT treatment characteristics

Characteristic	No.	%
Location of lesion	71	
Left upper lobe	20	28.2
Left lower lobe	10	14.1
Right upper lobe	21	29.6
Right middle lobe	7	9.9
Right lower lobe	13	18.3
Radiation modality	71	
Tomo	45	63.4
IMRT	26	36.6
Peripheral or central type	71	
Peripheral	54	76.1
Central	17	23.9
Dose‐total (Gy)	71	
Median(Range)	50(30‐70)	
BED (Gy)	71	
Median(Range)	100 (58‐180)	
≥100Gy	54	76.1
<100Gy	17	23.9
Fractionation	71	
10Gy × 5F	37	52.1
7Gy × 10F	17	23.9
5Gy × 10F	17	23.9
Lesion volume (mm^3^)	71	
Median(Range)	10.45 (3‐157)	
Efficacy evaluation for SBRT	71	
CR	5	7
PR	36	50.7
SD	23	32.4
PD	7	9.9

### 2.3 Follow‐up and evaluation

A follow‐up CT scan was performed every 3 months in the first 2 years, every 6 months in years 3‐5, and annually thereafter. Additional imaging, such as MR imaging or FDG‐PET, was also performed if clinically indicated. All patients were registered until death or loss to follow‐up. Curative effect was evaluated by Response Evaluation Criteria in Solid Tumors (RECIST) 1.1. LC was defined as no progressive disease of the pulmonary lesions within or at the margin of the PTV. Recurrences distant to the treated pulmonary lesions in the same lobe were not classified as local failure but as intrathoracic regional recurrence. PFS was defined as the time from SBRT initiation to progression (including local, regional, or distant progression) or death from any cause; OS was defined as time from SBRT initiation to death from any cause. Acute and late normal tissue toxicities were graded by using National Cancer Institute Common Terminology Criteria for Adverse Events (CTCAE), version 4.0.

### 2.4 Statistics

All statistical analyses were conducted using Statistical Package for Social Scientists (SPSS/Windows, Version 22.0, SPSS Inc, Chicago, USA). Descriptive statistics were used for categorical variables (frequency and percentage) and continuous variables (median and range). LC, PFS, and OS rates were analyzed using the Kaplan‐Meier method with 95% confidence intervals (CIs). Univariable and multivariable Cox regression analyses were performed to explore prognostic factors. The multivariable Cox regression analysis simultaneously included factors that had shown associations (*P* < 0.100) in the univariable Cox regression analyses, and variables based on their clinical significance according to previous literature reports. Cut‐off values of continuous variables were calculated using the receiver‐operating characteristic curve analyses. All tests were two‐sided and *P*‐values of <0.05 were considered statistically significant.

## 3 RESULTS

### 3.1 Patient characteristics

Between January 2012 and June 2018, a total of 75 consecutive oligometastatic NSCLC patients were prospectively studied. Excluding four patients with short follow‐up (<3 months), 71 eligible patients with complete follow‐up and clinical data were included in this retrospective study. Of the cohort, the median age was 67 years (33‐85 years) and the mean BMI was 22.65 (95%CI:19.4‐25.9). Among them, there were 19 males (26.9%) and 52 females (73.1%), and 29 patients (40.8%) were ever or current smokers. There were 15 patients with squamous carcinoma, and 56 patients with nonsquamous carcinoma. The median of CEA level before SBRT was 4.89 ng/mL (range:1‐632 ng/mL). Thirteen oligometastatic patients had primary tumors surgical resection previously. There were 37 patients receiving EGFR‐TKIs treatment, and 15 patients receiving chemotherapy before SBRT treatment, and there were 19 patients without treatment before SBRT. Additionally, 40, seven, two patients received first, second, third line treatment before SBRT, respectively. The median of time interval between systemic treatment and SBRT initiation was 6.2 months (range: 0‐57.4 months). There were 31, 6, 16, 4 and 12 patients with the extrapulmonary metastatic sites of bone, liver, brain, adrenal and other, respectively. A total of 15 patients had two lesions (including primary pulmonary lesions) in lung, so there were 86 lesions out of 71 patients in total (Table 1).

### 3.2 Treatment outcomes

**Figure 1:**
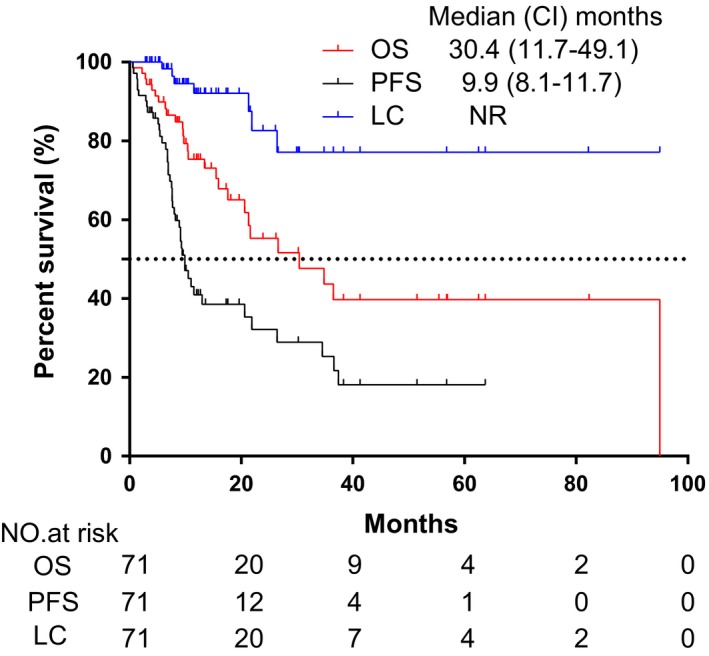
Kaplan‐Meier plot of OS, PFS, and LC in oligometastatic NSCLC patients with pulmonary lesions. OS, overall survival; PFS, progression‐free survival; LC, local control; NSCLC, non‐small cell lung cancer; CI, confidence interval; HR, hazard ratio

The median duration of follow‐up of 71 oligometastatic NSCLC patients in the study was 17.6 months (95%CI:11.2‐24.0 months). The median OS observed in this study was 30.4 months (95% CI:11.7‐49.1 months)0.1‐year, 2‐year, and 3‐year OS rates were 75.4%, 55.3%, and 54.4%, respectively (Figure 1).

The overall response rate (ORR) of the pulmonary lesions evaluated by RECIST 1.1 was complete for 7% (5/71), partial for 50.7% (36/71), stable for 32.4% (23/71), and progressive for 9.9% (7/71). A total of seven patients experienced in‐field local recurrence during follow‐up time. The median time to local failure was 12.7 months (95% CI:10.7‐14.7 months). LC rates were 92.1% after 1 year, 82.6% after 2 years, and 77.1% after 3 years, respectively (Figure 1).

For PFS, 43 patients (43/71, 60.6%) progressed during follow‐up time. Median PFS was 9.9 months (95% CI:8.1‐11.7 months). 1‐year, 2‐year, and 3‐year PFS rates were 41.0%, 32.1%, and 25.3%, respectively (Figure 1). The major progression pattern was distant failure, which occurred in 38 patients (38/71, 53.5%).

### 3.3 Adverse events

There was no case of CTCAE v 4.0 grade 4 to 5 toxicity. None of the patients who died had any evidence of treatment‐related toxicity. RP grade 1 (asymptomatic pulmonary changes) occurred in 10 (77.5%) patients. Three patients (4.2%) had grade 2 complaints, and three patients had grade 3 toxicity. Besides RP, one patient experienced bronchial stricture that was likely related to SBRT after completing treatment plan for 6 months. This patient was treated with SBRT for a central pulmonary lesions with prescription of 10Gy × 5F. Acute grade 1 and grade 2 hematologic toxicities occurred in six (8.5%) and one patients (1.4%), respectively; these included grade 1 leucopenia (n = 4), grade 2 leucopenia (n = 1), and grade 1 anemia (n = 2), respectively. Other toxicities included grade 2 fatigue (n = 4), grade 1 fatigue (n = 6), grade 1 nausea (n = 1), and grade 1 anorexia (n = 2). Patient‐and treatment‐related variables were evaluated by the logistic analysis model. No variables were significantly correlated with the RP.

### 3.4 Univariate and multivariate analysis

Results of univariate Cox regression analyses for PFS and OS are shown in Table 3. PFS was most significantly influenced by systemic treatment regimen before SBRT and age (*P* ≤ 0.01). Additionally, primary tumor histology, and time interval between systemic treatment and SBRT initiation also significantly influenced PFS (*P* < 0.05) (Table 3). Other factors such as gender, smoking, number of metastases, CEA level before SBRT, lesion size were not found to be prognostic for PFS. Regarding OS in univariate Cox regression analyses, neither age nor gender was found to be statistically significant. Adenocarcinoma showed a higher risk compared to other histologies. Additionally, systemic treatment regimen before SBRT influenced OS (Table 3). Whereas PS score, CEA level before SBRT, and number of metastases were not associated with OS benefit significantly. Regarding LC, no factor was found to be statistically significant by univariate Cox regression analyses.

**Table 3:** Univariate and multivariate analyses for the factors associated with PFS and OS

Factors	Univariate analysis of PFS (%)			Univariate analysis of OS (%)		
	HR	95%CI	*P*	HR	95%CI	*P*
Gender: male vs female	0.835	0.428‐1.633	0.599	1.222	0.512‐2.914	0.652
Age, y						
> 70 vs ≤70	0.353	0.165‐0.754	**0.007**	0.493	0.196‐1.241	0.133
ECOG PS score: <2 vs ≥2	0.789	0.332‐1.874	0.591	0.502	0.138‐1.826	0.296
BMI	1.036	0.935‐1.150	0.498	1.100	0.963‐1.256	0.160
Smoking: yes VS no	0.742	0.399‐1.378	0.344	0.693	0.315‐1.523	0.361
CEA level before SBRT, ng/mL	1.001	0.999‐1.004	0.174	1.001	0.998‐1.004	0.415
Lesion volume, mm^3^	1.003	0.995‐1.011	0.512	0.998	0.985‐1.011	0.303
Histology			**0.013**			0.076
Squamous carcinoma vs adenocarcinoma	0.361	0.150‐0.868	**0.023**	0.247	0.058‐1.054	0.059
Other vs adenocarcinoma	0.346	0.134‐0.893	**0.028**	0.407	0.121‐1.368	0.146
Number of metastases (all sites)			0.311			0.550
2 vs 1	1.345	0.636‐2.845	0.438	1.554	0.590‐4.092	0.372
3 or more vs 1	1.808	0.845‐3.873	0.127	1.684	0.625‐4.537	0.303
Systemic treatment regimen before SBRT			**0.002**			**0.039**
TKIs vs chemotherapy	0.381	0.181‐0.803	**0.011**	0.523	0.212‐1.290	0.159
None vs chemotherapy	0.196	0.079‐0.486	**0.000**	0.202	0.059‐0.693	**0.011**
Time interval between systemic treatment initiation and SBRT: >193 vs ≤193 days	2.030	1.107‐3.721	**0.022**	1.308	0.605‐2.826	0.495

The bold figures showed the statistically significant differences.

CI, confidence interval; HR, hazard ratio; OS, overall survival; PFS, progression‐free survival; SBRT, stereotactic body radiotherapy.

**Figure 2:**
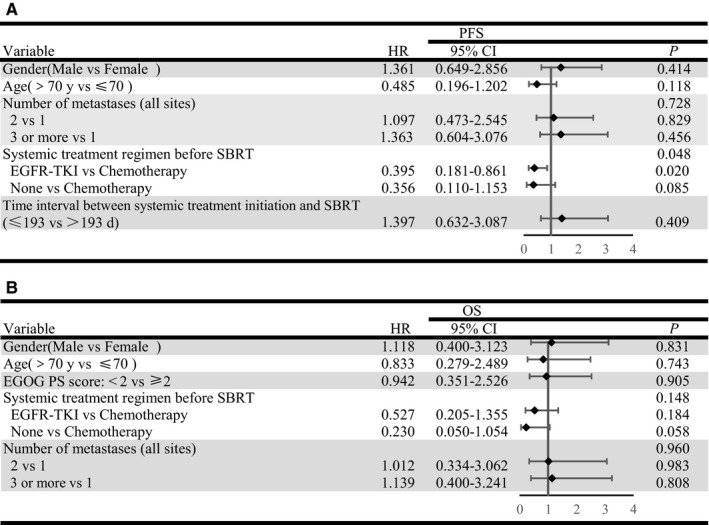
Multivariate analyses and forest plots indicating the independent prognostic factors of (A) PFS and (B) OS. PFS, progression‐free survival; OS, overall survival; HR, hazard ratio; CI, confidence interval

In order to balance the statistical significance and clinical significance, the multivariable Cox regression analyses simultaneously included factors that had shown associations (*P* < 0.100) in the univariable Cox regression analyses, and other variables based on their clinical significance that were reported to be associated with survival in previous studies. Moreover, as shown in univariate analyses, histology categaries and systemic treatment regimens presented a similar effect on survival. However histology strongly correlated with the systemic treatment regimens, which was confirmed by chi‐square test (*P* = 0.016). Consequently, systemic treatment regimen before SBRT rather than histology was included in the final multivariate Cox regression model. Results of multivariate Cox regression analyses for PFS and OS are shown in Figure 2A, B. Systemic treatment regimen before SBRT also had a significant impact on PFS (*P* < 0.05). Patients underwent chemotherapy were at higher risk of failure compared to those with EGFR‐TKIs therapy (*P* = 0.02) (Figure 2A).

### 3.5 Subgroup analyses

**Figure 3:**
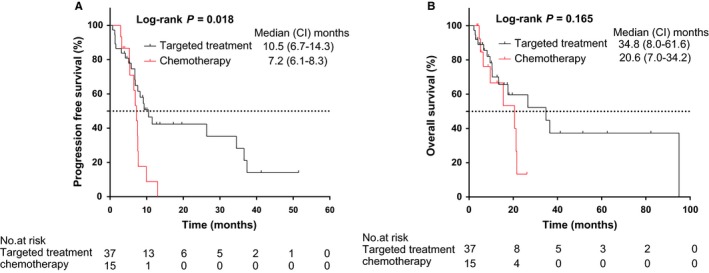
Log‐rank comparisons of all patients grouped on EGFR‐TKIs therapy vs chemotherapy before SBRT for (A) PFS and (B) OS. PFS, progression‐free survival; OS, overall survival; HR, hazard ratio; CI, confidence interval

**Figure 4:**
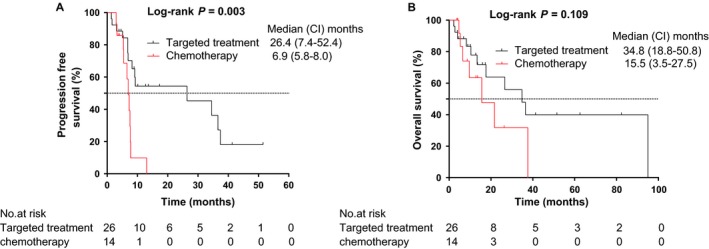
Kaplan‐Meier plot of (A) PFS and (B) OS in patients treated with first line treatment grouped on variable that different first systemic treatment regimens before SBRT. PFS, progression‐free survival; OS, overall survival; HR, hazard ratio; CI, confidence interval

According to the multivariate Cox regression analyses, it seemed that the use of EGFR‐TKIs was an independent prognostic factor of survival (Figure 2A). Moreover, we performed Log‐rank comparisons between EGFR‐TKIs therapy and chemotherapy to investigate the impact on PFS and OS (Figure 3). EGFR‐TKIs group showd a better outcomes than chemotherapy group, with PFS and OS being 10.9m vs. 7.2m and 38.4m vs. 20.6m respectively. The use of EGFR‐TKIs could significantly improve PFS (P < 0.05), but the survival benefit failed to expand to OS (P > 0.05). One possible reason was the confounding effects caused by subsequent treatments on OS. Hence, to further evaluate the impact of SBRT during first line systemic treatment on PFS and OS, the groups “≥second line treatment” and “without treatment before SBRT” were excluded from population, and survival curves of PFS and OS were performed grouped on variables of different first systemic treatment regimens (Figure 4). It was also confirmed that SBRT following chemotherapy presented a significantly shorter PFS and OS, compared to EGFR‐TKIs therapy (*P* < 0.05). It was shown that the median PFS and OS of first line EGFR‐TKIs therapy were 26.4 months (95%CI:7.4‐52.4months) and 34.8 months (95%CI:18.8‐50.8 months), respectively. The median PFS and OS of first line chemotherapy were 6.9 months (95%CI:5.8‐8.0months) and 15.5 months (95%CI:3.5‐27.5 months), respectively. It is noticeable that the median time interval between systemic treatment and SBRT initiation in the group in which patients received first line systemic treatment before SBRT was 6.3 months.

## 4 DISCUSSION

Historically, NSCLC patients with oligometastases treated with surgical resection suggested a more durable disease control.^19^, ^20^ The development of SBRT could be effectively used in the oligometastatic setting. Small prospective and retrospective studies have suggested both clinical efficacy and reasonable safety of SBRT for patients with oligometastases.^21^, ^22^, ^23^, ^24^ The existing literature of SBRT for pulmonary oligometastases reports 2‐year LC rates of 77.9%‐89.0% and 2‐year OS rates of 53.7%‐73.0%.^21^, ^25^, ^26^, ^27^, ^28^ Our study included 71 oligometastatic NSCLC patients treated with SBRT for pulmonary lesions between January 2012 and June 2018. It showed 2‐year LC rates of 82.6% and 2‐year OS rates of 55.3%. At the same time, we observed a low incidence of grade 3 toxicity, three cases of grade 3 toxicity involved RP in patients. Hence, similar clinical outcomes and reasonable safety of SBRT for oligometastatic NSCLC patients specific to pulmonary lesions also have been confirmed in our study.

A retrospective analysis of metastatic NSCLC patients treated with chemotherapy at the University of Colorado demonstrated that progression after first line chemotherapy most often occurred at known sites at baseline, rather than distant metastasis.^18^ Iyengar et al further complemented data in oligo‐progressive, that SBRT showed the potential for effective use as a salvage setting in shifting the pattern of failure from known sites to new sites.^29^ Small prospective and retrospective studies suggested that the outcomes of patients with oligometastatic stage IV NSCLC who received consolidative radiotherapy is more similar with the outcomes of patients with stage III disease.^29^, ^30^, ^31^, ^32^, ^33^, ^34^ Our results of oligometastatic NSCLC patients treated with SBRT are consistent with above studies. The major progression pattern was distant failure, which occurred in 38 patients (a total of 43 patients progressed, crude incidence, 88.4%). Moreover, comparing with a median PFS of 2‐4 months in stage IV NSCLC patients treated with maintenance chemotherapy alone,^35^ we reported a prolonged median PFS of 9.9 months (95% CI:8.1‐11.7 months) and median OS of 30.4 months (95% CI:11.7‐49.1 months), despite a broad range of patients with no restriction for oligometastatic disease were included in maintenance chemotherapy trials. Whereas even in the highly selective oligometastatic trials, the oligometastatic setting also did not show better outcomes when patients received maintenance chemotherapy alone. These trials typically have shown a respective PFS of 3.5 and 3.9 months by Iyengar^29^, ^33^ and Gomez.^34^

However, there were some patients treated with EGFR‐TKIs before SBRT in our study. It is well‐known that the use of TKIs might predict better outcomes. According to the multivariate Cox regression analysis, it seemed that systemic treatment regimen before SBRT was an independent prognostic factor of survival (Figure 2). Consequently, we further performed Log‐rank comparisons between EGFR‐TKIs therapy and chemotherapy to investigate the impact on survival (Figure 3). It was shown a median PFS of 10.9 months (95% CI:6.7‐14.3 months) and median OS of 34.8 months (95% CI:8.0‐61.6 months) in EGFR‐TKIs therapy group, and a median PFS of 7.2 months (95% CI:6.1‐8.3 months) and median OS of 20.6 months (95% CI:7.0‐34.2months) in chemotherapy group. The PFS and OS are measured from the initiation of SBRT rather than systemic treatment, so our results still showed remarkable outcomes. Whereas our study also found the use of EGFR‐TKIs just significantly influenced PFS (*P* < 0.05), but was not found to significantly influence OS (*P* > 0.05). It might be the subsequent treatments that influenced OS.

Consequently, we further evaluated the impact of first line treatment regimes on survival (Figure 4). It was shown that the median PFS and OS of first line chemotherapy group were 6.9 months (95%CI:5.8‐8.0months) and 15.5 months (95%CI:3.5‐27.5 months), respectively. The median time interval between first line systemic treatment initiation and SBRT was 6.3 months. There are some recently reported studies of oligometastatic NSCLC treated with radiotherapy following chemotherapy showing median PFS of 9.7‐11.2 months and median OS of 28.4 months ‐NR (Not reached).^29^, ^33^, ^34^, ^36^ Our PFS and OS of SBRT following first chemotherapy were consistent with that of these three studies. It was well known that the impact of chemotherapy is mainly on micrometastatic disease, but with limited durability of visible tumors. Our results also confirmed that SBRT could significantly prolong PFS for oligometastatic NSCLC patients after initial chemotherapy. However, data regarding the consolidative SBRT for oligometastatic stage IV NSCLC patients with EGFR mutation during first line EGFR‐TKIs therapy are sparse.^34^, ^37^, ^38^, ^39^ Our study showed that the median PFS and OS of first line EGFR‐TKI therapy group were 26.4 months (95%CI:7.4‐52.4months) and 34.8 months (95%CI:18.8‐50.8 months), respectively. Zhou et al^40^ performed a retrospective single‐institutional analysis of patients with oligometastatic stage IV EGFR‐mutant NSCLC. The study showed a median PFS of 20.6 months and a median OS of 40.9 months, obtained from EGFR‐TKIs therapy combined with local ablative therapy. Our results of SBRT were in line with it. Comparing with a median PFS of 8‐11 months in stage IV EGFR‐mutant NSCLC patients treated with EGFR‐TKI therapy alone,^41^, ^42^ our results suggested that SBRT had an important role on prolonging PFS of oligometastatic EGFR‐mutant NSCLC patients, although the sample size was small.

In conclusion, the findings of this study are as follows. First, SBRT for pulmonary lesions in oligometastatic NSCLC patients was a feasible and tolerable option. Second, systemic treatment regimen before SBRT was an independent prognostic factor of survival, and oligometastatic NSCLC patients with EGFR mutation were appropriate candidates for SBRT. Finally, delivery of SBRT for patients with oligometastatic NSCLC could significantly prolong PFS compared with historical controls, whether prior systemic treatment was chemotherapy or EGFR‐TKI therapy. Certainly, there are several limitations in our study; it is a retrospective single‐arm study in a single institution, which inevitably resulted in a selection bias. A more finely devised prospective and random study is needed to confirm the conclusion.
